# Maternal immunisation: What have been the gains? Where are the gaps? What does the future hold?

**DOI:** 10.12688/f1000research.15475.1

**Published:** 2018-11-01

**Authors:** Michelle L. Giles, Sushena Krishnaswamy, Euan M. Wallace

**Affiliations:** 1 Ritchie Centre, Department of Obstetrics and Gynaecology, Monash University, Melbourne, VIC, Australia; 2 Monash Infectious Diseases, Monash Health, Melbourne, VIC, Australia; 3 Safer Care Victoria, Victorian Government, Melbourne, VIC, Australia

**Keywords:** vaccination, pregnancy, women, respiratory syncytial virus, group B streptococcus, influenza

## Abstract

The vaccination of pregnant women has enormous potential to protect not only mothers from vaccine-preventable diseases but also their infants through the passive acquisition of protective antibodies before they are able to themselves acquire protection through active childhood immunisations. Maternal tetanus programmes have been in place since 1989, and as of March 2018, only 14 countries in the world were still to reach maternal neonatal tetanus elimination status. This has saved hundreds of thousands of lives. Building on this success, influenza- and pertussis-containing vaccines have been recommended for pregnant women and introduced into immunisation programmes, albeit predominantly in resource-rich settings. These have highlighted some important challenges when additional immunisations are introduced into the antenatal context. With new vaccine candidates, such as respiratory syncytial virus (RSV) and group B streptococcus (GBS), on the horizon, it is important that we learn from these experiences, identify the information gaps, and close these to ensure safe and successful implementation of maternal vaccines in the future, particularly in low- and middle-income countries with a high burden of disease.

## Introduction

In 2015, the Sustainable Development Goals (SDGs) were launched to replace the Millennium Development Goals previously set, in 2000, by the United Nations to guide the eradication of poverty, hunger, illiteracy, and disease ^1^. The third SDG is to ensure healthy lives and promote well-being at all ages. An important target of this SDG is to end preventable deaths of newborns and children under five years of age by 2030. All countries should be aiming to reduce neonatal mortality to 12 per 1000 live births or lower and under-five mortality to 25 per 1000 live births or lower ^2^. If every country were to achieve these SDG targets for child survival by 2030, then 10 million more children would survive to age five. Half of these will be additional newborn babies surviving past one month of age.

In 2016, the worldwide mortality rate for children under five years of age was 41 per 1000 live births. This is half the worldwide rate in 1990 ^3^. The first 28 days of life constitute the most vulnerable period for children. In 2015, the global neonatal mortality rate was 19 per 1000 live births, a fall from 31 per 1000 live births in 2000. Along with prematurity and intrapartum-related complications, infectious diseases—particularly pneumonia, sepsis, and respiratory illness—are leading causes of death in children ^4^. Vaccination against infectious diseases has had a key role in improving child health ^5, 6^. However, most childhood vaccinations start at six weeks of age and many diseases require more than one dose of vaccine to confer adequate protection. This leaves newborn infants vulnerable in their first months of life. Vaccination of the pregnant mother (maternal immunisation) has emerged as a potential strategy to reduce the morbidity and mortality of very young infants during this vulnerable period.

Maternal immunisation provides transient immunity to the newborn by the transplacental transfer of maternal immunoglobulin G (IgG) antibodies. This begins around 13 weeks’ gestation and increases throughout pregnancy such that the majority of antibody transfer occurs in the last trimester of pregnancy ^7, 8^. In the context of maternal immunisation, this is an important concept as preterm infants may not have the opportunity for the same protection if vaccinations are either recommended or given late in pregnancy. Antibodies can also be transferred to newborns via breast milk. For example, IgA antibody to pertussis toxin is present in breast milk following maternal immunisation ^9^. Although the highest level has been reported in colostrum, pertussis-specific IgA has been detected for eight weeks in breast milk ^9^.

The World Health Organization (WHO) and national policy makers from different countries recommend routine tetanus and influenza vaccination for pregnant women and, in specific settings, vaccination for pertussis, hepatitis (A and B), yellow fever, meningococcus, pneumococcus, and polio ^10, 11^. In addition to these, new vaccines are on the horizon to address other causes of neonatal morbidity and mortality, such as respiratory syncytial virus (RSV) and group B streptococcus (GBS). In this article, we summarise the gains made thus far in maternal immunisation, the gaps that remain, and the goals and opportunities for maternal immunisation to improve maternal and child health.

## What have been the gains?

One of the greatest success stories of maternal immunisation in some countries has been the effective elimination of maternal and neonatal tetanus through maternal vaccination. In 1988, the WHO estimated that 787,000 newborns died of neonatal tetanus, stimulating the 42nd World Health Assembly the following year to call for the elimination of neonatal tetanus by 1995 ^12^. To achieve this, low-resource countries have implemented tetanus toxoid vaccination programmes to pregnant women. By March 2018, while 14 countries have yet to reach maternal and neonatal tetanus elimination status, there has been a 96% reduction in neonatal mortality from tetanus—over 750,000 lives saved—compared with the late 1980s ^12^. The majority of this gain has been achieved by maternal immunisation ^12^.

A more recent example of gains afforded by maternal immunisation relates to pertussis infection. Hospitalisation and infant mortality due to pertussis disproportionately affect children less than six months of age ^13^. This is likely because children require at least two doses of pertussis-containing vaccine before they are adequately protected, and, in most vaccination programmes, the first immunisation is not given until two months of age. To address this, maternal immunisation has been recommended as a strategy in many resource-rich countries, including the US since 2011, the UK since 2012, and Australia since 2015 ^14– 16^.

In 2012, in response to high rates of disease in infants under three months of age and an increase in pertussis-related deaths, the UK’s Department of Health recommended a vaccination programme including a pertussis-containing vaccine for all women in the third trimester of pregnancy ^15^. Evaluation of the effectiveness of this programme showed that it reduced pertussis infection in infants less than eight weeks of age by 90% ^17^. Various studies conducted in the UK, the US, and Spain have now confirmed more than 90% effectiveness of maternal pertussis vaccination in preventing laboratory-confirmed pertussis in infants less than two to three months of age ^18– 21^. Vaccine effectiveness against infant pertussis-related death is estimated at 95% ^18^. Since the introduction of the maternal pertussis immunisation programme in the UK, there have been 16 infant deaths between 2013 and 2015, compared with 14 infant deaths alone in 2012. Of the 16 infants who died after introduction of the programme, 14 were babies whose mothers were not vaccinated. In the case of both of the remaining infants who died, the mother was vaccinated less than 10 days prior to delivery ^18^. This highlights a key implementation issue related to maternal immunisation: identifying the optimal timing of administration of maternal vaccine to maximise transplacental passage of maternal antibodies. In relation to pertussis, research data support the clinical findings cited above that vaccination early in the third trimester, or even possibly in the second trimester, is most likely to achieve a protective level of antibodies in the baby ^22, 23^. Both tetanus and pertussis provide examples of how maternal immunisation programmes, when successfully implemented, can prevent vaccine-related disease in infants and, in the case of tetanus, elimination.

## Where are the gaps?

Despite the successes of tetanus and pertussis vaccination, many key gaps in the field of maternal immunisation remain. For example, influenza vaccination has been recommended for pregnant women since the 1960s ^24^. This is because influenza infection is associated with more severe disease in pregnant women. In 2012, the WHO Strategic Advisory Group for Experts on Immunisation recommended pregnant women as the most important risk group to benefit from inactivated seasonal influenza vaccination ^25^. Despite this global recommendation and evidence for efficacy in the prevention of influenza in pregnant women and their babies ^26^, not all countries recommend or are able to implement maternal influenza vaccination programmes. A review of national influenza immunisation worldwide policies, undertaken by the WHO and UNICEF, showed that, of the 115 WHO member states that had an influenza immunisation policy, less than half included pregnant women ^27^. Inclusion of pregnant women in a national policy was more likely in high- or upper middle-income countries ^27^. This highlights an important challenge in maternal immunisation: how do we expand immunisation programmes beyond maternal tetanus in low- and middle-income countries to include additional vaccines with potential benefits to pregnant women or the infant or both? It is particularly challenging because in the world regions with the greatest burden of newborn deaths, Southern Asia and sub-Saharan Africa ^28^, less than half of all pregnant women have access to adequate pregnancy care ^29^.

Furthermore, for the successful delivery of effective maternal vaccination programmes, beyond strengthening of basic health services and skilled personnel, there are other factors that need to be considered before implementation of any new maternal vaccine, including knowledge of pathogen-specific epidemiology, country-specific burden of disease data among pregnant women and their newborns, implementation costs, and vaccine effectiveness and safety ^30^. Indeed, perceived safety concerns have been identified as a key barrier to vaccine uptake by pregnant women ^31^. Even in countries with a fully funded programme, uptake of influenza vaccine during pregnancy remains low ^32– 36^.

This is disappointing because, in 2011, the WHO’s Strategic Advisory Group of Experts on Immunisation tasked the Global Advisory Committee on Vaccine Safety (GACVS) to review the evidence on safety of vaccinations in pregnant women, including influenza, tetanus toxoid, rubella, meningococcal, oral polio, and yellow fever vaccine. The GACVS report included the outcomes of maternal morbidity and mortality, miscarriage/stillbirth, prematurity, small size for gestational age, and congenital anomalies. There was no evidence of any adverse outcome—maternal or perinatal—associated with vaccination ^37^. Since the publication of the GACVS report, there have been five systematic reviews of influenza vaccine safety in pregnancy ^38– 42^. All reviews concluded that for either mother or foetus there were no safety concerns associated with the use of influenza vaccines ^38– 42^. This highlights an important gap in our understanding. Why do women and health-care providers still cite safety concerns as an important reason for not receiving influenza vaccine during pregnancy despite this evidence?

One reason may be related to the language and content of product information provided by the influenza vaccine manufacturers. A review by Proveaux *et al*. reported on 96 separate influenza vaccines and found that 21% of these included language suggesting that official recommendations should be “considered” ^43^. Half of the products suggested that users consult a health-care provider to determine whether the product should be given during pregnancy, and only 10% suggested use during pregnancy ^43^. In addition, a subsequent study of 141 maternal health-care providers from 49 countries in all six WHO regions suggested that health-care providers perceive product information as contradicting WHO and national immunisation recommendations and that this could affect their decision to recommend the vaccine to pregnant women ^44^.

Importantly, not only has there been no safety signal identified in all the systematic reviews undertaken in relation to influenza vaccination during pregnancy, but there are actually data suggesting a statistically significant benefit to the newborn in terms of reduced preterm birth ^45– 48^ and stillbirth ^49^. There is also evidence for protection against laboratory-confirmed influenza for the first 6 months of life for the newborn ^50, 51^. A randomised controlled trial in pregnant women compared influenza vaccine with placebo and reported a vaccine efficacy of 43% against all-cause acute lower respiratory tract infection and hospitalisation in the first six months of life and no difference in rates of preterm birth and low birth weight between the vaccinated and unvaccinated groups ^50^. An additional randomised trial comparing influenza vaccine with placebo in pregnancy had an overall efficacy of 30% in reducing laboratory-confirmed influenza infections in infants less than six months of age ^51^. In this randomised controlled trial, maternal immunisation reduced the rate of low birth weight by 15% but did not modify the rate of small-for-gestational-age birth. The differences in reported non-specific protective effects such as on preterm and small-for-gestational-age birth may be impacted by time-dependent variables which are inadequately controlled for in studies ^52^. This requires further evaluation as reducing preterm birth, particularly in low- and middle-income countries, will contribute significantly to achieving the SDGs by 2030.

## What does the future hold?

RSV and GBS are two important causes of neonatal morbidity and mortality ^53, 54^ that are attractive vaccine candidates for maternal immunisation programmes.

RSV is an important cause of lower respiratory tract illness in infants globally and is responsible for one third of deaths due to lower respiratory tract infection in children less than one year of age ^55^. Infants under six months of age are particularly susceptible, so as is the case with tetanus, pertussis, and influenza—maternal immunisation may be an effective strategy to confer protection during this vulnerable period. As with any maternal vaccine, the magnitude of benefit to the mother, foetus, and newborn may differ. In evaluating a new maternal vaccine, it is important to measure the potential maternal benefit along with the benefit to the child. The maternal effects of RSV infection during pregnancy are only just beginning to be understood. A recent publication by Chu *et al*. described the clinical presentation and birth outcomes of RSV infection in pregnancy in Nepal ^56^. Of the cases observed, 50% sought medical care, and of those infected during pregnancy, 29% delivered preterm births ^56^. It is important to note, however, that the absolute number of cases in this report is small (only 14 cases detected overall). In contrast, a recent publication from South Africa ^57^ did not report any association between maternal RSV infection and adverse pregnancy outcomes. Post-partum infection however, was associated with concurrent infection in 52% of infants ^57^.

Currently, an RSV vaccine candidate for pregnant women is undergoing a phase III clinical trial (ClinicalTrials.gov identifier: NCT02624947). The trial investigators aim to recruit 8,618 women and administer either vaccine or placebo in the third trimester of pregnancy. The primary outcome is RSV-proven lower respiratory tract infection with hypoxemia in the infant. Effectiveness and safety have yet to be established.

The goals of a maternal programme against RSV would be to prevent infant death and hospitalisation, prevent or reduce the severity of lower respiratory tract illness in young infants, reduce transmission in the household and community, reduce antibiotic usage for treatment of lower respiratory tract illness, and potentially reduce maternal effects of RSV during pregnancy ^58^. However, there are many important pieces of information required to fully understand the potential magnitude of benefit that an RSV vaccine may offer. Importantly, RSV burden of disease data, particularly mortality and morbidity in low- and middle-income countries, is essential and is currently lacking. In addition, successful implementation will be possible only if the vaccine is affordable and both health-care providers and pregnant women understand the benefits and can be reassured in relation to the safety of the vaccine.

GBS is an important cause of neonatal sepsis and meningitis, especially in the first three months of life. In 2015, worldwide, an estimated 205,000 infants developed early-onset disease (defined as occurring at or within 24 hours of birth through day 6 after birth) and 11,400 infants had late-onset disease (between 7 and 90 days of life). There were an estimated 90,000 deaths in infants less than three months of age and 33,000 cases of invasive GBS disease in pregnant or post-partum women. It has been estimated that a maternal GBS vaccine with 80% efficacy and 90% coverage could prevent 107,000 stillbirths and infant deaths ^54^. More specifically, models have estimated that with a vaccine efficacy of 70% and coverage equal to the proportion of pregnant women with at least four antenatal visits, maternal GBS immunisation would prevent one third of GBS cases and deaths in Uganda and Nigeria, 42 to 43% in Guinea-Bissau, and 55 to 57% in Ghana ^59^.

The most common current strategy to reduce neonatal sepsis is screening for GBS in pregnant women and administration of intrapartum antibiotics to those who are colonised ^60^. It has been shown to reduce early-onset neonatal GBS sepsis but has no impact on late-onset GBS infection ^60^. In addition, the strategy of screening and antibiotics is often challenging in settings where women infrequently attend for antenatal care and where access to diagnostic testing and intravenous antibiotics during labour is limited. These challenges make a GBS vaccine approach appealing.

GBS candidate vaccines have been investigated in phase I and II clinical trials ^61– 67^. These trials have used bivalent and trivalent vaccines (serotypes Ia, Ib, and III). More recently, vaccine manufacturers are focusing on pentavalent vaccines covering the five GBS serotypes which account for more than 90% of invasive neonatal disease. An important data requirement with candidate vaccines is information on effectiveness, particularly in low- and middle-income countries, using clinical endpoints. This may be challenging when designing future GBS vaccine trials given the need for a large sample size to adequately power the study and for robust surveillance and diagnostic systems to adequately confirm endpoints. Therefore, immunological correlates of protection may need to be considered as surrogate endpoints for licensure of GBS vaccines ^68^. Despite these challenges, establishing effectiveness and safety is essential prior to recommending any new maternal vaccine and must remain a priority as candidate GBS vaccines are developed.

## What more needs to be done?

Maternal immunisation, though not a new concept, is gaining momentum as an important, safe, and effective strategy to prevent infant morbidity and mortality in addition to providing direct protection to the mother. Embracing this and applying the principles learned from implementation of other maternal vaccines to other infectious diseases such as RSV and GBS hold enormous promise, particularly in countries with the highest rate of childhood mortality. Maternal immunisation may contribute significantly to achieving the SDG target to end preventable deaths of newborns and children under five years of age by 2030. However, increased resources and effort need to be invested in understanding disease burden, particularly in low- and middle-income countries so the populations that stand to benefit the most from these strategies can be identified. Clearly, vaccine effectiveness and safety data are crucial; however, as has been seen with other maternal vaccinations, unless there is adequate education of women and health-care providers and consideration given to optimal implementation strategies, the maximal benefit from maternal vaccination programmes will not be achieved.

## Abbreviations

GACVS, Global Advisory Committee on Vaccine Safety; GBS, group B streptococcus; RSV, respiratory syncytial virus; SDG, Sustainable Development Goal; WHO, World Health Organization
